# Supplementary figures and images for: Preventing the Distortion of CoO6 Octahedra of LiCoO2 at High-Voltage Operation of Lithium-Ion Battery: An Organic Surface Reinforcement
Source: Polymers (Basel). 2023 May 6;15(9):2211. doi: 10.3390/polym15092211 (PMC10181088; doi:10.3390/polym15092211)

# **Supporting Material**

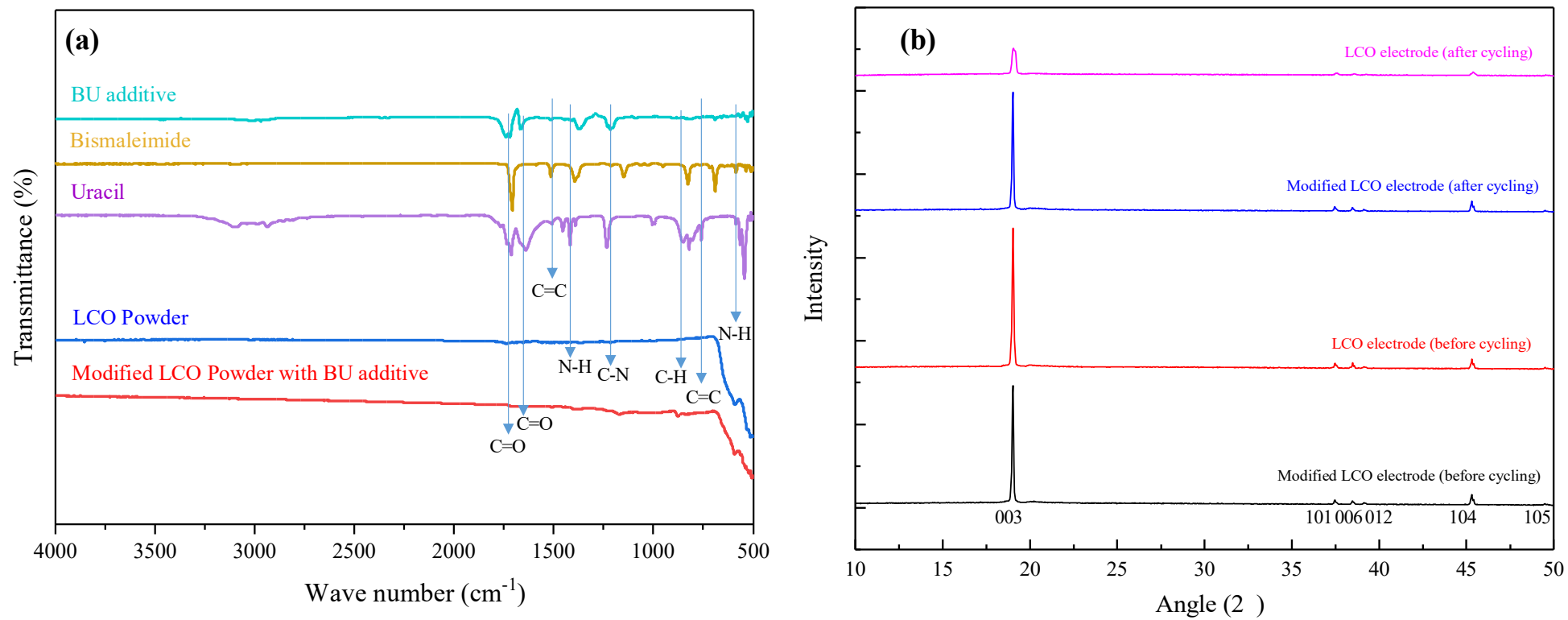

**Figure S1** (a) FTIR spectra of powders and (b) XRD pattern of electrodes.

Supplement: Supplementary file 1 [file polymers-15-02211-s001.zip › polymers-2228622-supplementary.pdf]
